# On Lactate of Zinc in Epilepsy

**Published:** 1856-10

**Authors:** M. Herpin


### *On Lactate of Zinc in Epilepsy.* By M. Herpin.

(Bull. Gen. de Therap. Nov. 1855.)

M. Herpin points out the fallacy of deductions from cases treated *en masse* by any remedy, without classifying the cases, and taking the prognosis into consideration. He divides cases of epilepsy into three groups. 1. Where the prognosis is *favorable.* This embraces cases in which there have been less than 100 attacks. 2. *Little favorable* cases, where there have been from 100 to 500 attacks. 3. *Unfavorable* cases, where there have been above 500 attacks. The duration of the affection, together with the age and sex of the subject, also influence the prognosis. All things being equal in respect to the number of fits, the most recent cases are the most favorable. Under five months' duration, the chances of recovery are twice as great as from five months to a year. After ten years, success is rare. Of all ages, old age is the most favorable; then youth and infancy; and least of all, adult age. In M. Herpin's hands there have been twice as many failures with males than with females. Adult men are most unfavorable subjects. To apply this sort of division to the cases treated by lactate of zinc: —of 41 epileptics, the treatment was only sufficiently advanced in 35 for any decision as to its effect being arrived at. Of these 35, 15 were favorable cases, 12 little favorable, and 8 unfavorable. Of the 8 unfavorables, 2 have improved to an extent which militates strongly in favor of the remedy. Of the 12 little favorable cases, in 2 children, aged respectively eight years and twenty-one months, the fits were suppressed; and a remarkable amelioration took place in one man. Of the 15 favorable cases, 4, in which various other remedies had failed, were influenced by the lactate; one of these had suffered 90 attacks in fifteen years and a half; a second, aged forty-four years, had symptoms of commencing general paralysis; a third, aged four or five years, had a hydrocephalic head; and a fourth, which had lasted three years and a half, was otherwise favorable. In 6 of the remaining 11, the attacks were suppressed; and of the 5 others, 3 have had the intervals so much prolonged, as to afford hope of complete cure on continuance of the remedy; the remaining two were amended. The remedy was given for a period of from five or six to twelve months.—*Med. Chir. Review.*

